# Correlation between Slug transcription factor and miR-221 in MDA-MB-231 breast cancer cells

**DOI:** 10.1186/1471-2407-12-445

**Published:** 2012-10-02

**Authors:** Elisabetta Lambertini, Andrea Lolli, Federica Vezzali, Letizia Penolazzi, Roberto Gambari, Roberta Piva

**Affiliations:** 1 Department of Biomedical and Specialty Surgical Sciences, University of Ferrara, Ferrara, 44121, Italy; 2 Department of Life Sciences and Biotechnology, University of Ferrara, Ferrara, 44121, Italy

**Keywords:** Slug, miR-221, Epithelial mesenchymal transition, Breast cancer

## Abstract

**Background:**

Breast cancer and its metastatic progression is mainly directed by epithelial to mesenchymal transition (EMT), a phenomenon supported by specific transcription factors and miRNAs.

**Methods:**

In order to investigate a possible correlation between Slug transcription factor and miR-221, we performed Slug gene silencing in MDA-MB-231 breast cancer cells and evaluated the expression of genes involved in supporting the breast cancer phenotype, using qRT-PCR and Western blot analysis. Chromatin immunoprecipitation and wound healing assays were employed to determine a functional link between these two molecules.

**Results:**

We showed that Slug silencing significantly decreased the level of miR-221 and vimentin, reactivated Estrogen Receptor α and increased E-cadherin and TRPS1 expression. We demonstrated that miR-221 is a Slug target gene, and identified a specific region of miR-221 promoter that is transcriptionally active and binds the transcription factor Slug “in vivo”. In addition, we showed that in Slug-silenced cells, wich retained residual miR-221 (about 38%), cell migration was strongly inhibited. Cell migration was inhibited, but to a less degree, following complete knockdown of miR-221 expression by transfection with antagomiR-221.

**Conclusions:**

We report for the first time evidence of a correlation between Slug transcription factor and miR-221 in breast cancer cells. These studies suggest that miR-221 expression is, in part, dependent on Slug in breast cancer cells, and that Slug plays a more important role than miR-221 in cell migration and invasion.

## Background

Epithelial cancers such as breast carcinomas and their metastatic progression are mainly directed by a phenomenon referred to as epithelial to mesenchymal transition (EMT) [1,2]. As well described in several reviews, EMT is supported by the same transcription factors (TFs) including ZEB factors and the Snail family of zinc finger proteins both during embryonic development and the metastatic cascade [1,3-5]. In addition, specific microRNAs (miRNAs) including miR-206, miR-221/222, miR-200, miR-141, miR-203, miR-130a, have been shown to regulate EMT [6-11].

Mounting evidence indicates that the acquisition of an aggressive cancer phenotype through EMT, as well as other cellular events, may be understood by evaluating the regulatory interplay between TFs and miRNAs [12,13]. Therefore, recent studies have investigated the interactions among specific miRNAs, TFs and target genes associated with this phenomenon. Direct evidence of these circuits in EMT is still little. Some specific networks have been described including miR-203 – Snai1 [14], a self-reinforcing loop miR-1/miR-200 via Slug [15], miR-200/miR-192 – p53 [16], miR-221/222 – TRPS1 [17], p53/miR-34 axis [18], and ZEB/miR-200 [19].

To investigate the key regulatory networks underlying EMT in breast cancer, we evaluated a potential correlation between Slug (SNAI2) transcription factor and miR-221. The ability of miR-221 and Slug to promote EMT and induce invasiveness in breast cancer cell lines has been documented, but crosstalk between these molecules has not been characterized [3,17,20].

Slug is a member of the Snail family of zinc-finger transcription factors, and, together with Snail (SNAI1), acts as a master regulator of EMT. Various studies over the past several years have documented the involvement of Slug in human cancers including leukemias [21], osteosarcoma [22], esophageal carcinomas [23], and breast cancers [3,24], where Slug expression is strongly correlated with the loss of E-cadherin. Multiple lines of evidence suggest that Slug can be considered a marker of malignancy as well as an attractive target for therapeutic modulation of invasiveness in the treatment of specific cancers [25-28].

miR-221 is often overexpressed in aggressive cancers, increases cell proliferation and protects cancer cells against different apoptotic stimuli [29-31]. Recently, the expression level of miR-221 has been significantly associated with Estrogen Receptor alpha (ERα) status in breast cancer, and several studies have demonstrated that miR-221 directly targets ERα [9,32,33]. Breast tumors from patients with high miR-221 plasma levels tend to be ERα-negative, more aggressive and show poorer clinical outcomes than ERα positive cancers [34]. In addition, ERα signaling has been correlated with Slug, and at least two different mechanisms showed that ERα decreases Slug expression [35-37].

In this study, we knocked down Slug and miR-221 in ERα-negative breast cancer cells, MDA-MB-231. We determined a functional correlation between these two molecules demonstrating “in vivo” interaction between Slug and miR-221. Rescue experiments with ectopic expression of miR-221, analysis of the expression of genes involved in breast cancer phenotype, and wound healing assay, suggested that the largest contribution to the invasion ability of the cells and their aggressive phenotype comes from Slug rather than miR-221.

## Methods

### Cell culture

Human breast cancer cell lines MDA-MB-231 and MDA-MB-436 were cultured in Dulbecco’s modified Eagle medium-High Glucose (DMEM-HG) (Euroclone S.p.a., Milan, Italy), supplemented with 10% Fetal Calf Serum (FCS) (Euroclone), 2 mM L-glutamine and 100 U/ml penicillin-streptomycin.

### Transfections

Breast cancer cells were transfected with 30 nM siRNA against Slug (Invitrogen, Carlsbad, CA) [38], 30 nM antagomiR-221, 50 nM pre-miR-221 precursor (named miR-221 mimic) (Ambion Life Technologies, Grand Island, NY), a non-relevant siRNA (si-Scr) (Medium GC Stealth RNAi Negative Control Duplex, Invitrogen), a non-relevant (miR-Scr) mimic and a non-relevant antagomiR (antagomiR-Scr) (Ambion Life Technologies, Grand Island, NY). For all transfections Lipofectamine RNAiMAX (Invitrogen) was used, following the manufacturer’s instructions. In brief, cells were plated the day before transfections in 12-well plates. Transfected cells were grown up to 6 days in a 37°C incubator with 5% CO_2_. Total RNA and proteins were extracted, and stored at −80°C for subsequent quantitative RT-PCR or Western Blot measurements. Each treatment used at least triplicate samples.

### RNA extraction

Total RNA including miRs was extracted from breast cancer cell lines using an RNeasy Mini Kit (Qiagen, Hilden, Germany) according to the manufacturer’s instruction and as previously described [39]. Total RNA was used for reverse-transcription and stored at −80°C. Briefly, cDNA was synthesized from total RNA (500 ng) in a 10 μl reaction volume using the TaqMan MicroRNA Reverse Transcription Kit (Applied Biosystems). The reactions were incubated first at 16°C for 30 min and then at 42°C for 30 min followed by inactivation at 85°C for 5 min.

### Quantitative real-time PCR for miRNA and mRNA quantification

Quantification of miR-221 and miR-222 was performed using TaqMan MicroRNA Assays (Applied Biosystems), followed by detection with the CFX96^TM^ PCR detection system (Bio-Rad, Hercules, CA). The TaqMan MicroRNA Assay for U6 snRNA (assay ID: 001973; Applied Biosystems) was used to normalize the relative abundance of miR-221 and miR-222. For quantification of Slug, E-cadherin, ERα and TRPS1 mRNAs and pri-miR-221 the appropriate TaqMan probes were purchased from Applied Biosystems using GAPDH reference gene for normalization. Relative expression was calculated using the comparative ΔΔCT method and the change in miRNA or mRNA expression was calculated as fold-change. All reactions were performed in triplicate. The experiment was repeated at least three times.

### Western blotting

For western blot analysis, the cells were washed twice with ice-cold PBS and cell lysates were prepared as previously reported [39]. Then, 20 μg of each sample were electrophoresed on a 12% SDS-polyacrylamide gel. The proteins were then transferred onto an Immobilon-P PVDF membrane (Millipore, Billerica, MA). After blocking with PBS-0.05% Tween 20 and 5% dried milk, the membrane was probed with the following antibodies: Slug (L40C6) from Cells Signaling Technology (Danvers, CA, USA), ERα (sc-544), E-cadherin (sc-7870), Vimentin (sc-7558) and p53 (sc-126) from Santa Cruz Biotechnology (Santa Cruz, CA)**.** After washing with PBS-Tween, the membranes were incubated with peroxidase-conjugated anti-rabbit antibody (1:50000) or anti-mouse (1:2000) (Dako, Glostrup, Denmark) in 5% non-fat milk. Immunocomplexes were detected using Supersignal West Femto Substrate (Pierce, Rockford, IL). Anti-IP3K was used to confirm equal protein loading.

### Viability analysis (calcein-AM uptake assay)

Viability assay was performed as described previously [40]. For propidium iodide and calcein analysis the cells were visualized under a fluorescence microscope (Nikon, Optiphot-2, Nikon corporation, Japan) using the filter block for fluorescein. Dead cells were stained in red, whereas viable ones appeared in green.

### Cell cycle analysis

Cell cycle analysis was performed using fluorescence-activated cell sorting (FACS). Briefly, MDA-MB-231 cells were collected 72 hours after transfection and stained with 25 μg/mL of propidium iodide (Roche Molecular Biochemicals, Indianapolis, IN) in phosphate-buffered saline containing 0.1% bovine serum albumin, 0.05% of Triton X-100, and 50 μg/mL of RNase A. Analysis were carried out using FACS Scan (Becton Dickinson, NJ).

### Cell proliferation assay

For growth curves analysis an equal number of cells (approximately 3 x 10^4^) were seeded into 24-well plates. Twenty-four hours after transfection, the cells were harvested and counted by trypan blue exclusion method every day up to three days and at day 6.

### Scratch wound assay

Forty-eight-hours after transfection a vertical wound was created in the MDA-MB-231 cell layer using a 20-μL pipette tip. Images were captured at designated times (0 and 24 hours) to assess the rate of gap closure.

### Chromatin immunoprecipitation (ChIP) assay

Chromatin immunoprecipitation (ChIP) assays were performed with the ChIP assay kit (Upstate Biotechnology, Lake Placid, NY) as previously described [38]. Briefly, MDA-MB-231 cells were grown to 70% confluency in DMEM supplemented with 10% FBS. Cross-linking was performed with 1% formaldehyde at 37°C for 10 min, the cells were washed in ice-cold PBS, and suspended in SDS lysis buffer supplemented with 1× protease inhibitor cocktail (Roche Molecular Biochemicals), for 10 min on ice. Samples were sonicated, diluted 10-fold in dilution buffer, and precleared with 80 μl of salmon sperm DNA-coated protein A-agarose beads; the supernatant was used directly for immunoprecipitation with anti-Slug, (sc-10436), anti-acetyl-H3 (sc-56616) or rabbit Ig λ chain control antibody (sc-33134) (Santa Cruz Biotechnology, INC) overnight at 4°C. Immunocomplexes were mixed with 80 μl of DNA-coated protein A-agarose beads followed by incubation for 1 h at 4°C. Beads were collected and sequentially washed 3 times with 1 ml each of the following buffers: low salt wash buffer (0.1% SDS, 1% Triton X-100, 2 mM EDTA, 20 mM Tris–HCl pH 8.1, 150 mM NaCl), high salt wash buffer (0.1% SDS, 1% Triton X-100, 2 mM EDTA, 20 mM Tris–HCl pH-8.1, 500 mM NaCl), LiCl wash buffer (0.25 mM LiCl, 1% IGEPAL-CA630, 1% deoxycholic acid, 1 mM EDTA, 10 mM Tris-pH 8.1), and TE buffer. The immunocomplexes were eluted twice by adding a 250 μl aliquot of a freshly prepared solution of 1% SDS, 0.1 M NaHCO_3_ and the cross-linking reactions were reversed by incubation at 65°C for 4 hrs. Further, the samples were digested with proteinase K (10 mg/ml) at 42°C for 1 hour and DNA was purified in 50 μL of Tris–EDTA with a PCR purification kit (Qiagen, Valencia, CA) according to the manufacturer’s instructions. For PCR analysis, aliquots of chromatin before immunoprecipitation were saved (Input). PCR was performed to analyze the presence of DNA precipitated by Slug-specific antibody, and by using specific primers to amplify fragments of the miR-221 and TRPS1 gene promoters. Each PCR reaction was performed with 5 μl of the bound DNA fraction or 2 μl of the Input. The PCR was performed as follows: preincubation at 95°C for 5 min, 30 cycles of 1 min denaturation at 95°C, 1 min annealing at the primers temperature, and 1 min at 72°C, with one final incubation at 72°C for 5 min. No-antibody negative control was included in each experiment.

### Statistical analysis

Data are presented as means ± SEM. For qRT-PCR and cell cycle analysis assays, statistical significance was analyzed by unpaired Student’s t test. p-values ≤ 0.05 were considered statistically significant.

## Results and discussion

### Correlation between Slug and miR-221 expression

**Figure 1:**
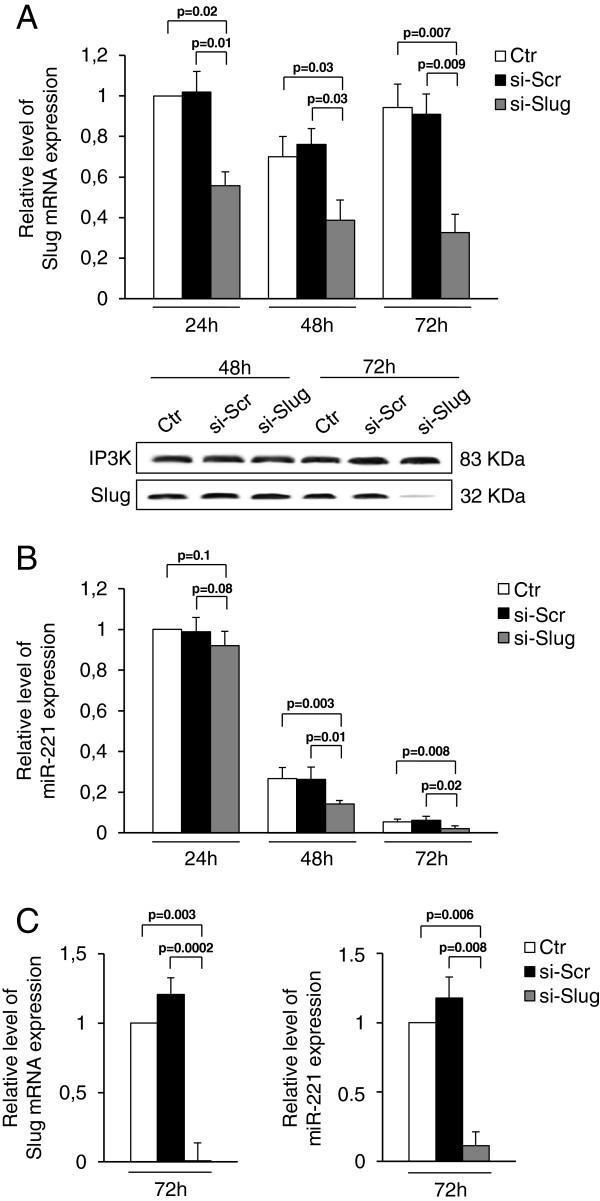
**Effect of Slug knockdown on miR-221 expression in breast cancer cells.** MDA-MB-231 (**A, B**) and MDA-MB-436 (**C**) breast cancer cells were transfected with 30nM si-Slug molecule or a non-relevant siRNA (si-Scr). Slug and miR-221 expression was determined at RNA level at three different times (24 h, 48 h, 72 h), and revealed by quantitative RT-PCR analysis. RT-PCR results were calculated using the ΔΔCt method and data are presented as fold change respect to control untreated cells (Ctr 24 h for MDA-MB-231, and Ctr 72 h for MDA-MB-436). Results represent means ± SEM of three independent experiments. p-values ≤ 0.05 were considered statistically significant. In panel A, Slug expression investigated at protein level and revealed by Western Blot, is reported. IP3K was used as loading control.

The ability of miR-221 and Slug to promote EMT in breast cancer cell lines, led us to investigate a potential correlation between these molecules. Consistent with previous observations, we confirmed that miR-221 and Slug are highly expressed in breast cancer cells such as MDA-MB-231 cell line (Figure 1A and B), and are associated with an aggressive phenotype. However, as demonstrated by time course experiments, miR-221 progressively decreases as cell culture proceeds (Figure 1B). This is not surprising since a large number of miRNAs shows distinct expression patterns that are often fluctuating as a consequence of their multi-functional roles [41,42]. Nevertheless, it is important to underline that although evidence of a role for miRNAs in cell differentiation is growing, the role of miRNAs in cell proliferation remains largely unexplored. For what concerns the expression of Slug, whose field of activity is more limited with respect to miR-221, its levels were unchanged during the culture period. We then examined the effect of Slug knockdown on miR-221 expression. The efficiency of small interfering RNA targeting Slug (si-Slug), was confirmed by qRT-PCR and Western blot. As shown in Figure 1A, Slug mRNA levels appreciably decreased after 24 hours, and Slug protein was almost completely abolished after 72 hours. Interestingly, si-Slug, but not a scrambled siRNA, significantly decreased miR-221 expression by 6% Figure 1B. In another ERα-negative breast cancer cell line, MDA-MB-436, Slug knockdown has the same effects (Figure 1C), strengthening the hypothesis that the presence of Slug is required for miR-221 expression. As the expression of these two molecules seems to be particularly correlated with the aggressive phenotype, we focused on MDA-MB-231 that are tumorigenic and highly metastatic compared to MDA-MB-436 that are non-tumorigenic and moderately metastatic [43,44].

**Figure 2:**
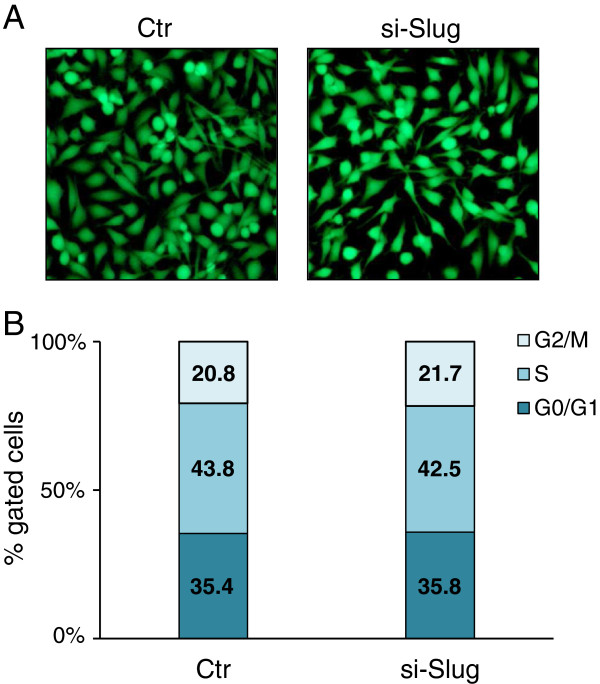
**Effect of Slug knockdown on cell cycle and viability.** MDA-MB-231 cells were transfected with si-Slug molecule and collected 72 hours after transfection. (**A**) Viability was determined by double staining assay with Calcein-AM and propidium iodide. Fluorescence photomicrographs (4X magnification) are representative merged images showing the presence of green fluorescence (calcein-AM)-labelled live cells and the absence of red fluorescence (PI)-labelled dead cells. (**B**) Cells were subjected to fluorescence-activated cell sorting analysis, and the relative G0/G1, S, and G2/M compartments calculated. Percentages of cells in each compartment are means of two independent experiments.

The effect of Slug silencing and the resulting decrease of miR-221 levels was evaluated on cell growth and viability using a Calcein-AM staining and flow cytometry analysis 72 h after treatment. As shown in Figure 2A, the viability of cells which have been transfected with siSlug was unaffected. These data were confirmed by flow cytometry assay. No statistically significant differences in the cell percentage were detected between Slug-silenced cells and control cells in different phases of cell cycle (Figure 2B).

### Recruitment of Slug at the miR-221-222 locus

**Figure 3:**
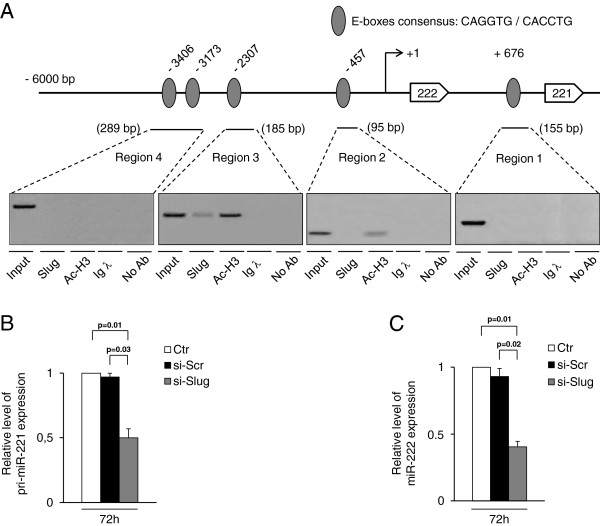
***In vivo *recruitment of Slug protein at the miR-221-222 locus.** (**A**) The localization of predicted Slug consensus binding sites (5'-CAGGTG-3' or 5'-CACCTG-3') in the human miR-222/221 locus region is indicated with grey ovals. Protein-DNA complexes were *in vivo* formaldehyde-cross linked in MDA-MB-231. Chromatin fragments were subjected to immunoprecipitation with antibodies against endogenous Slug and Acetyl Histone H3 (Ac-H3). A negative control using nonspecific normal rabbit antibody against Ig λ chain was also included. After cross-link reversal, the coimmunoprecipitated DNA was amplified by PCR using the primers pairs spanning the reported regions of miR-221 promoter (PCR amplicons are indicated by horizontal bars). Aliquots of chromatin taken before immunoprecipitation were used as Input positive controls whereas chromatin eluted from immunoprecipitation lacking antibody was used as no antibody control (No Ab). All experiments were repeated at least three times and representative images shown. MDA-MB-231 cells were transfected with 30 nM si-Slug molecule or a non-relevant siRNA (si-Scr). Pri-miR-221 (**B**) and miR-222 (**C**) expression levels were determined at RNA level after 72 h of treatment, and revealed by quantitative RT-PCR analysis. RT-PCR results were calculated using the ΔΔCt method and data are presented as fold change respect to untreated cells (Ctr). Results represent means ± SEM of three independent experiments. p-values ≤ 0.05 were considered statistically significant.

The involvement of Slug in miR-221 regulation was further investigated by chromatin immunoprecipitation (ChIP) assay. The human genomic DNA sequences belonging to the entire 5^′^ regulatory region of miR-222/221 locus have been analyzed for the presence of putative Slug binding sites (E-box motifs, 5^′^-CANNTG-3^′^) [3,38], using Transcription Element Search Software (TESS) for transcription factor search and MatInspector 7.4 programs. As shown in Figure 3, five potential candidates to mediate Slug regulatory function in the miR-221 promoter are present in the region. We performed ChIP analysis to determine whether endogenous Slug transcription factor is recruited at the identified E boxes consensus sequences. Four chromatin sub-regions were analyzed for E-boxes occupancy by PCR revealing that region 3 was specifically involved in the interaction, whereas no chromatin was immunoprecipitated by the regions 1, 2 and 4 (Figure 3A). When the ChIP assay was performed against acetylated histone 3 (Anti-AcH3), a colocalization with Slug in region 3 was detected, indicating that the identified region 3 of miR-221 promoter is transcriptionally active and is involved in the binding of transcription factor Slug. As shown in Figure 3B, following Slug silencing, the level of miR-221/222 primary transcript significantly decreased (by 50%), further demonstrating that miR-221 is a Slug target gene and is transcriptionally regulated by Slug. At the same time, the concomitant decrease of miR-222 expression levels, after Slug silencing, demonstrated that Slug is involved in the regulation of the entire miR-222/221 locus (Figure 3C).

### Effect of Slug silencing on specific gene expression

**Figure 4:**
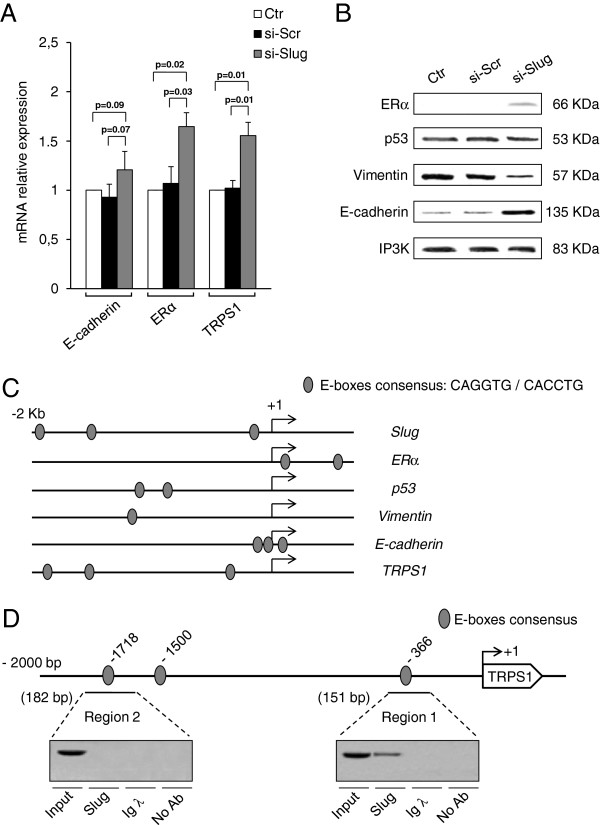
**Effect of Slug knockdown on the expression of specific genes.** MDA-MB-231 cells were transfected with si-Slug molecule or a non-relevant siRNA (si-Scr). (**A**) E-cadherin, ERα, TRPS1 expression was determined at mRNA level, and revealed by quantitative RT-PCR analysis. Data are represented as fold change respect to control sample (Ctr) for each gene analysed. Results represent means ± SEM of three independent experiments. p-values ≤ 0.05 were considered statistically significant. (**B**) ERα, p53, Vimentin, and E-cadherin expression was determined at protein level, and revealed by Western Blot. (**C**) Analysis of the 2 Kb in size promoter region of Slug, ERα, p53, Vimentin, E-cadherin and TRPS1 genes. Predicted E-boxes consensus-binding site are indicated with grey ovals. (**D**) Slug is recruited at TRPS1 promoter *in vivo*. The localization of predicted Slug consensus binding sites (5'-CAGGTG-3' or 5'-CACCTG-3') in the human TRPS1 promoter is reported. Protein-DNA complexes were *in vivo* formaldehyde-cross linked in MDA-MB-231 cells. Chromatin fragments were subjected to immunoprecipitation with antibody against endogenous Slug. A negative control using nonspecific normal rabbit antibody against Ig λ chain was also included. After cross-link reversal, the coimmunoprecipitated DNA was amplified by PCR using the primers pairs spanning the reported regions of TRPS1 promoter (PCR amplicons are indicated by horizontal bars). Aliquots of chromatin taken before immunoprecipitation were used as Input positive controls whereas chromatin eluted from immunoprecipitation lacking antibody was used as no antibody control (No Ab). All experiments were repeated at least three times and representative images shown.

There is evidence that many genes involved in promoting metastasis are highly expressed both in miR-221 expressing cells and in cells with high expression levels of Slug. In addition, in the same cells, the expression of many genes with a critical role in suppressing tumor growth and metastasis was found to be repressed. To further evaluate the correlation between Slug and miR-221, we analyzed the expression of some of these genes involved in supporting the breast cancer phenotype in Slug-silenced MDA-MB-231 cells (Figure 4). Expression levels of E-cadherin [45], ERα [36], and GATA family transcriptional repressor TRPS1 (tricho-rhino-phalangeal syndrome type 1) [17], were investigated by quantitative RT-PCR (Figure 4A). ERα, p53, vimentin and E-cadherin expression was analyzed by Western blot (Figure 4B). Notably, si-Slug silencing reactivated ERα, increased E-cadherin and TRPS1 and decreased vimentin. On the contrary, altering Slug expression levels did not affect p53 expression. Change of gene expression was not observed in si-Scr oligomer transfected cells. These results indicate that, at the molecular level, breast cancer cells with a mesenchymal phenotype, such as MDA-MB-231, when transfected with siRNA against Slug, decrease the EMT program, reactivating an epithelial phenotype. The involvement of Slug in the regulation of ERα, p53, vimentin and E-cadherin, and in the activation of its own promoter by a direct binding to E-box, has been demonstrated by several lines of evidence [37,46-49]. This is confirmed by the presence of E boxes in the 5^′^ regulatory region of ERα, p53, vimentin, and E-cadherin genes using TESS software (Figure 4C). The same analysis also revealed that the promoter of TRPS1 gene contains putative Slug binding sites. ChIP analysis performed on the entire sequence identified a specific involvement of region 1, but not region 2, in the recruitment of Slug at TRPS1 promoter in vivo (Figure 4D). This suggests a direct role of Slug in the regulation of the expression of TRPS1 gene, demonstrating for the first time a link between these two molecules.

The decrease of ERα and TRPS1 expression is a marker of poor clinical outcome in breast cancers. Therefore, although further investigations are required to better understand the correlation among Slug, miR-221, TRPS1 and ERα, nevertheless, removal of Slug and the consequent down-regulation of miR-221 and reactivation/increase of ERα and TRPS1, may be taken into account for the treatment of ERα-negative breast cancer.

**Figure 5:**
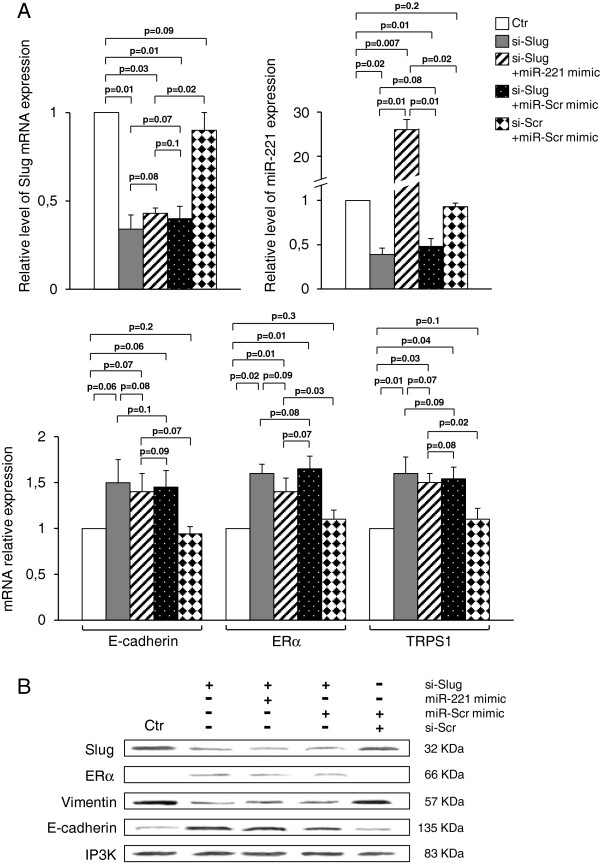
**Effect of miR-221 overexpression on the expression of specific genes.** MDA-MB-231 cells were transfected with si-Slug molecule alone, si-Slug in combination with miR-221 mimic or a non-relevant miR (miR-Scr) mimic, a non-relevant siRNA (si-Scr) in combination with miR-Scr mimic. (**A**) Slug**,** miR-221**,** E-cadherin, ERα, TRPS1 expression was determined at RNA level, and revealed by quantitative RT-PCR analysis. RT-PCR results were calculated using the ΔΔCt method. Data are represented as fold change respect to control sample (Ctr). Results represent means ± SEM of three independent experiments. p-values ≤ 0.05 were considered statistically significant. (**B**) Slug, ERα, Vimentin, and E-cadherin expression was determined at protein level, and revealed by Western Blot. Ten micrograms of whole cell lysates were assayed on a 12% SDS-PAGE, and the proteins were visualized using Supersignal Femto Substrate (Pierce). IP3K was used as loading control.

In order to estimate the contribution of miR-221 to Slug-dependent gene regulation, Slug-silenced cells were transfected with miR-221 mimic to evaluate a possible rescue effect of miR-221 overexpression. As shown in Figure 5, the miR-221 overexpression did not restore Slug expression and did not balance si-Slug-mediated up-regulation of E-cadherin, ERα and TRPS1. No significant difference of gene expression was observed in the cells transfected with a combination of si-Scr and miR-Scr mimic, in comparison to untreated cells. In addition, the presence of miR-Scr mimic in si-Slug transfected cells did not affect the effect of Slug silencing.

These results suggest that miR-221 down-modulation has not major implications in the phenotype arising from Slug silencing, as ectopic miR-221 expression cannot fully rescue it. In addition, this simultaneous modulation of Slug and miR-221 suggests that silence of Slug could significantly protect cells from progression towards an aggressive phenotype or metastatic stimuli that, in this case, are represented by miR-221 overexpression.

### Slug is required for cellular invasion and migration

To better characterize the correlation between Slug and miR-221 at the functional level, the effects of their knockdown on the invasive potential of MDA-MB-231 cells were evaluated using the scratch-wound healing assay that is usually employed to determine in vitro migratory ability of the cells.

**Figure 6:**
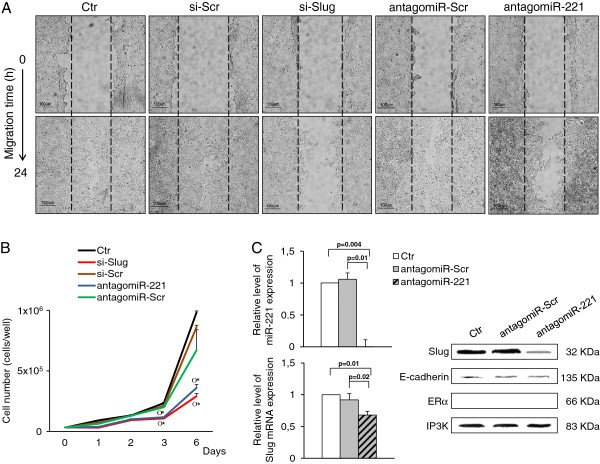
**Effect of Slug and miR-221 knockdown on MDA-MB-231 cell migration ability.** (**A**) Cells were transfected with 30nM si-Slug, a non-relevant siRNA (si-Scr), antagomiR-221 or a non-relevant antagomiR (antagomiR-Scr). Twenty-four hours after transfection, cells monolayer were scratch wounded with a 20-μl pipet tip (0 h), and observed over the indicated time periods, 0 and 24 hours (4x magnification). Scale bar =100 μm. (**B**) Proliferation curves of MDA-MB-231 cells exposed to si-Slug, si-Scr, antagomiR-221 and antagomiR-Scr up to six days. Statistical analysis was performed si-Slug or antagomiR-221 treated cells versus untreated cells (Ctr) (*), and si-Slug or antagomiR-221 treated cells versus si-Scr or antagomiR-Scr respectively (o); p ≤ 0.05. (**C**) Slug and miR-221 RNA levels were analysed by quantitative RT-PCR after antagomiR-221 or antagomiR-Scr treatment, and results were calculated using the ΔΔCt method. Data are presented as fold difference respect to control untreated cells (Ctr). Results represent means ± SEM of three independent experiments. p-values ≤ 0.05 were considered statistically significant. The expression of Slug, E-cadherin, ERα proteins was also analyzed by Western Blot. IP3K was used as loading control.

As revealed by a representative scratch assay (Figure 6A), 24 h after cell monolayers were wounded, control cells (untreated or scrambled cells) had almost completely filled the cleared area. On the contrary, Slug-repressed cells, with a residual amount of miR-221 of approximately 38%, showed strongly impaired cell migration. Therefore, gap closure in Slug repressed cells was significantly reduced because migration from the border of the wound was very slow.

Interestingly, complete knockdown of miR-221 expression by transfection with antagomiR-221, significantly attenuated the gap closing in MDA-MB-231 cells, but not as much as that observed in Slug-repressed cells. These findings confirm the role of miR-221 in the cell invasive potential, and its involvement in promoting the EMT phenotype [7,8], but suggest that the largest contribution to the migratory ability comes from Slug rather than miR-221.

Data from the wound healing assay may in part be explained with the change of cells growth ability, and in part with the change of expression of specific genes. As expected, Slug or miR-221 knocked down cells significantly reduced their proliferation rate compared to control cells (untreated or scrambled cells) (Figure 6B). At the same time, we found that miR-221 knockdown causes a significant but not sufficient decrease of Slug expression (Figure 6C). In fact, residual Slug mRNA (68%) only slightly decreased the level of Slug protein, and consequently E-cadherin expression was almost unaffected, as revealed by Western blot analysis. This molecular evidence supports the higher ability of miR-221-repressed MDA-MB-231 cells to close the wounded area compared to Slug-silenced cells, strengthening our hypothesis that Slug is indeed linked to cancer cell migration and invasion more than miR-221. In addition, as previously reported [50], we confirm that restoration of ERα could not be achieved by miR-221 knockdown in ERα mRNA-negative cell lines such as MDA-MB-231 (Figure 6C), supporting the notion that ERα is a direct target of miR-221 at the translation level.

Furthermore, data from miR-221 knockdown suggest that unlike Slug, probably one of its negative regulators could be a miR-221 target. While further investigations on a possible Slug / miR-221 circuit are needed, our data suggest that Slug is preferable to miR-221 as potential target to obtain inhibition or slowing down of EMT and metastasis.

## Conclusions

Taken together, the results presented here provide for the first time evidence of a correlation between Slug transcription factor and miR-221 in MDA-MB-231 breast cancer cells. However, considering the complexity of EMT phenomenon, further experiments are needed to explore the possible Slug / miR-221 circuit, especially to understand regulatory interactions with potential unknown factors acting as molecular mediators inside the loop. This report suggests that miR-221 is, in part, dependent on Slug in breast cancer cells, and that Slug plays a more important role than miR-221 in cell migration and invasion. Therefore, our evidence may be useful for developing therapeutical approaches for poor prognosis breast cancers.

## Competing interests

The authors declare that they have no competing interests.

## Authors’ contributions

RP and EL organized the study. AL, LP, FV and EL carried out the cell culture and molecular studies, and participated in the data analysis. RG participated in the design and coordination of the study. RP, EL, AL and RG contributed to the interpretation of the results. All authors read and approved the final manuscript. The authors thank dr. Brya Grace Matthews for English language corrections.

## Pre-publication history

The pre-publication history for this paper can be accessed here:

http://www.biomedcentral.com/1471-2407/12/445/prepub

## References

[B1] Nieto MA The ins and outs of the epithelial to mesenchymal transition in health and disease Annu Rev Cell Dev Biol 2011 27 347 376 10.1146/annurev-cellbio-092910-154036 21740232

[B2] Vincent-Salomon A Thiery JP Host microenvironment in breast cancer development: epithelial-mesenchymal transition in breast cancer development Breast Cancer Res 2003 5 2 101 106 10.1186/bcr578 12631389 PMC154156

[B3] De Herreros AG Peiró S Nassour M Savagner P Snail family regulation and epithelial mesenchymal transitions in breast cancer progression J Mammary Gland Biol Neoplasia 2010 15 2 135 147 10.1007/s10911-010-9179-8 20455012 PMC2930904

[B4] Barrallo-Gimeno A Nieto MA The Snail genes as inducers of cell movement and survival: implications in development and cancer Development 2005 132 14 3151 3161 10.1242/dev.01907 15983400

[B5] Thiery JP Acloque H Huang RYJ Nieto MA Epithelial-mesenchymal transitions in development and disease Cell 2009 139 5 871 890 10.1016/j.cell.2009.11.007 19945376

[B6] Gregory PA Bracken CP Bert AG Goodall GJ MicroRNAs as regulators of epithelial-mesenchymal transition Cell Cycle 2008 7 20 3112 3118 10.4161/cc.7.20.6851 18927505

[B7] Guttilla IK Adams BD White BA ERα, microRNAs, and the epithelial-mesenchymal transition in breast cancer Trends Endocrinol Metab 2012 23 2 73 82 10.1016/j.tem.2011.12.001 22257677

[B8] Wright JA Richer JK Goodall GJ microRNAs and EMT in mammary cells and breast cancer J Mammary Gland Biol Neoplasia 2010 15 2 213 223 10.1007/s10911-010-9183-z 20499142

[B9] Howe EN Cochrane DR Richer JK The miR-200 and miR-221/222 microRNA families: opposing effects on epithelial identity J Mammary Gland Biol Neoplasia 2012 17 1 65 77 10.1007/s10911-012-9244-6 22350980 PMC4561555

[B10] Burk U Schubert J Wellner U Schmalhofer O Vincan E Spaderna S Brabletz T A reciprocal repression between ZEB1 and members of the miR-200 family promotes EMT and invasion in cancer cells EMBO Rep 2008 9 6 582 589 10.1038/embor.2008.74 18483486 PMC2396950

[B11] Wellner U Schubert J Burk UC Schmalhofer O Zhu F Sonntag A Waldvogel B Vannier C Darling D Zr Hausen A Brunton VG Morton J Sansom O Schüler J Stemmler MP Herzberger C Hopt U Keck T Brabletz S Brabletz T The EMT activator ZEB1 promotes tumorigenicity by repressing stemness-inhibiting microRNAs Nat Cell Biol 2009 11 12 1487 1495 10.1038/ncb1998 19935649

[B12] Reshmi G Sona C Pillai MR Comprehensive patterns in microRNA regulation of transcription factors during tumor metastasis J Cell Biochem 2011 112 9 2210 2217 10.1002/jcb.23148 21503963

[B13] Wang J Haubrock M Cao KM Hua X Zhang CY Wingender E Li J Regulatory coordination of clustered microRNAs based on microRNA-transcription factor regulatory network BMC Syst Biol 2011 5 199 10.1186/1752-0509-5-199 22176772 PMC3262773

[B14] Moes M Le Béchec A Crespo I Laurini C Halavatyi A Vetter G Del Sol A Friederich E A novel network integrating a miRNA-203/SNAI1 feedback loop which regulates epithelial to mesenchymal transition PLoS One 2012 7 4 e35440 10.1371/journal.pone.0035440 22514743 PMC3325969

[B15] Liu YN Yin JJ Abou-Kheir W Hynes PG Casey OM Fang L Yi M Stephens RM Seng V Sheppard-Tillman H Martin P Kelly K MiR-1 and miR-200 inhibit EMT via slug-dependent and tumorigenesis via slug-independent mechanisms Oncogene 2012 in press 10.1038/onc.2012.58 PMC7580497 22370643

[B16] Kim T Veronese A Pichiorri F Lee TJ Jeon YJ Volinia S Pineau P Marchio A Palatini J Suh SS Alder H Liu CG Dejean A Croce CM p53 regulates epithelial-mesenchymal transition through microRNAs targeting ZEB1 and ZEB2 J Exp Med 2011 208 5 875 883 10.1084/jem.20110235 21518799 PMC3092351

[B17] Stinson S Lackner MR Adai AT Yu N Kim HJ O’Brien C Spoerke J Jhunjhunwala S Boyd Z Januario T Newman RJ Yue P Bourgon R Modrusan Z Stern HM Warming S de Sauvage FJ Amler L Yeh RF Dornan D TRPS1 targeting by miR-221/222 promotes the epithelial-to-mesenchymal transition in breast cancer Sci Signal 2011 4 177 ra41 10.1126/scisignal.2001538 21673316

[B18] Kim NH Kim HS Li XY Lee I Choi HS Kang SE Cha SY Ryu JK Yoon D Fearon ER Rowe RG Lee S Maher CA Weiss SJ Yook JI A p53/miRNA-34 axis regulates Snail1-dependent cancer cell epithelial-mesenchymal transition J Cell Biol 2011 195 3 417 433 10.1083/jcb.201103097 22024162 PMC3206336

[B19] Bracken CP Gregory PA Kolesnikoff N Bert AG Wang J Shannon MF Goodall GJ A double-negative feedback loop between ZEB1-SIP1 and the microRNA-200 family regulates epithelial-mesenchymal transition Cancer Res 2008 68 19 7846 7854 10.1158/0008-5472.CAN-08-1942 18829540

[B20] Shah MY Calin GA MicroRNAs miR-221 and miR-222: a new level of regulation in aggressive breast cancer Genome Med 2011 3 8 56 10.1186/gm272 21888691 PMC3238182

[B21] Mancini M Petta S Iacobucci I Salvestrini V Barbieri E Santucci MA Zinc finger transcription factor slug contributes tothe survival advantage of chronic myeloid leukemia cells Cell Signal 2010 22 8 1247 1253 10.1016/j.cellsig.2010.04.002 20388540

[B22] Guo Y Zi X Koontz Z Kim A Xie J Gorlick R Holcombe RF Hoang BH Blocking Wnt/LRP5 signaling by a soluble receptor modulates the epithelial to mesenchymal transition and suppresses met and metalloproteinases in osteosarcoma Saos-2 cells J Orthop Res 2007 25 7 964 971 10.1002/jor.20356 17318900

[B23] Jethwa P Naqvi M Hardy RG Hotchin NA Roberts S Spychal R Tselepis C Overexpression of Slug is associated with malignant progression of esophageal adenocarcinoma World J Gastroenterol 2008 14 7 1044 1052 10.3748/wjg.14.1044 18286686 PMC2689407

[B24] Côme C Magnino F Bibeau F De Santa Barbara P Becker KF Theillet C Savagner P Snail and slug play distinct roles during breast carcinoma progression Clin Cancer Res 2006 12 18 5395 5402 10.1158/1078-0432.CCR-06-0478 17000672

[B25] Vitali R Mancini C Cesi V Tanno B Mancuso M Bossi G Zhang Y Martinez RV Calabretta B Dominici C Raschellà G Slug (SNAI2) down-regulation by RNA interference facilitates apoptosis and inhibits invasivegrowth in neuroblastoma preclinical models Clin Cancer Res 2008 14 14 4622 4630 10.1158/1078-0432.CCR-07-5210 18628477 PMC7199277

[B26] Howard EW Camm KD Wong YC Wang XH E-cadherin upregulation as a therapeutic goal in cancer treatment Mini Rev Med Chem 2008 8 5 496 518 10.2174/138955708784223521 18473938

[B27] Mimeault M Batra SK Functions of tumorigenic and migrating cancer progenitor cells in cancer progression and metastasis and their therapeutic implications Cancer Metastasis Rev 2007 26 1 203 214 10.1007/s10555-007-9052-4 17273942

[B28] Hotz B Arndt M Dullat S Bhargava S Buhr HJ Hotz HG Epithelial to mesenchymal transition: expression of the regulators snail, slug, and twist in pancreatic cancer Clin Cancer Res 2007 13 16 4769 4776 10.1158/1078-0432.CCR-06-2926 17699854

[B29] Galardi S Mercatelli N Giorda E Massalini S Frajese GV Ciafrè SA Farace MG miR-221 and miR-222 expression affects the proliferation potential of human prostate carcinoma cell lines by targeting p27Kip1 J Biol Chem 2007 282 32 23716 23724 10.1074/jbc.M701805200 17569667

[B30] Pineau P Volinia S McJunkin K Marchio A Battiston C Terris B Mazzaferro V Lowe SW Croce CM Dejean A miR-221 overexpression contributes to liver tumorigenesis Proc Natl Acad Sci USA 2010 107 1 264 269 10.1073/pnas.0907904107 20018759 PMC2806773

[B31] Felicetti F Errico MC Bottero L Segnalini P Stoppacciaro A Biffoni M Felli N Mattia G Petrini M Colombo MP Peschle C Carè A The promyelocytic leukemia zinc finger-microRNA-221/-222 pathway controls melanoma progression through multiple oncogenic mechanisms Cancer Res 2008 68 8 2745 2754 10.1158/0008-5472.CAN-07-2538 18417445

[B32] Di Leva G Gasparini P Piovan C Ngankeu A Garofalo M Taccioli C Iorio MV Li M Volinia S Alder H Nakamura T Nuovo G Liu Y Nephew KP Croce CM MicroRNA cluster 221–222 and estrogen receptor alpha interactions in breast cancer J Natl Cancer Inst 2010 102 10 706 721 10.1093/jnci/djq102 20388878 PMC2873185

[B33] Rao X Di Leva G Li M Fang F Devlin C Hartman-Frey C Burow ME Ivan M Croce CM Nephew KP MicroRNA221/222 confers breast cancer fulvestrant resistance by regulating multiple signaling pathways Oncogene 2011 30 9 1082 1097 10.1038/onc.2010.487 21057537 PMC3342929

[B34] Zhao R Wu J Jia W Gong C Yu F Ren Z Chen K He J Su F Plasma miR-221 as a predictive biomarker for chemoresistance in breast cancer patients who previously received neoadjuvant chemotherapy Onkologie 2011 34 12 675 680 10.1159/000334552 22156446

[B35] Ye Y Xiao Y Wang W Yearsley K Gao JX Barsky SH ERalpha suppresses slug expression directly by transcriptional repression Biochem J 2008 416 2 179 187 10.1042/BJ20080328 18588516 PMC2584332

[B36] Ye Y Xiao Y Wang W Yearsley K Gao JX Shetuni B Barsky SH ERalpha signaling through slug regulates E-cadherin and EMT Oncogene 2010 29 10 1451 1462 10.1038/onc.2009.433 20101232

[B37] Dhasarathy A Kajita M Wade PA The transcription factor snail mediates epithelial to mesenchymal transitions by repression of estrogen receptor-alpha Mol Endocrinol 2007 21 12 2907 2918 10.1210/me.2007-0293 17761946 PMC2668600

[B38] Torreggiani E Lisignoli G Manferdini C Lambertini E Penolazzi L Vecchiatini R Gabusi E Chieco P Facchini A Gambari R Piva R Role of Slug transcription factor in human mesenchymal stem cells J Cell Mol Med 2012 16 4 740 751 10.1111/j.1582-4934.2011.01352.x 21645238 PMC3822845

[B39] Lambertini E Lisignoli G Torreggiani E Manferdini C Gabusi E Franceschetti T Penolazzi L Gambari R Facchini A Piva R Slug gene expression supports human osteoblast maturation Cell Mol Life Sci 2009 66 22 3641 3653 10.1007/s00018-009-0149-5 19756381 PMC11115484

[B40] Penolazzi L Tavanti E Vecchiatini R Lambertini E Vesce F Gambari R Mazzitelli S Mancuso F Luca G Nastruzzi C Piva R Encapsulation of mesenchymal stem cells from Wharton’s jelly in alginate microbeads Tissue Eng Part C Methods 2010 16 1 141 155 10.1089/ten.tec.2008.0582 19402785

[B41] Chen Y Gelfond J McManus LM Shireman PK Temporal microRNA expression during in vitro myogenic progenitor cell proliferation and differentiation: regulation of proliferation by miR-682 Physiol Genomics 2011 43 10 621 630 10.1152/physiolgenomics.00136.2010 20841498 PMC3110887

[B42] Ciaudo C Servant N Cognat V Sarazin A Kieffer E Viville S Colot V Barillot E Heard E Voinnet O Highly dynamic and sex-specific expression of microRNAs during early ES cell differentiation PLoS Genet 2009 5 8 e1000620 10.1371/journal.pgen.1000620 19714213 PMC2725319

[B43] Cailleau R Olivé M Cruciger QV Long-term human breast carcinoma cell lines of metastatic origin: preliminary characterization In Vitro 1978 14 11 911 915 10.1007/BF02616120 730202

[B44] Yang X Welch DR Phillips KK Weissman BE Wei LL KAI1, a putative marker for metastatic potential in human breast cancer Cancer Lett 1997 119 2 149 155 10.1016/S0304-3835(97)00273-5 9570365

[B45] Baranwal S Alahari SK Molecular mechanisms controlling E-cadherin expression in breast cancer Biochem Biophys Res Commun 2009 384 1 6 11 10.1016/j.bbrc.2009.04.051 19379710 PMC2700729

[B46] Kajita M McClinic KN Wade PA Aberrant expression of the transcription factors snail and slug alters the response to genotoxic stress Mol Cell Biol 2004 24 17 7559 7566 10.1128/MCB.24.17.7559-7566.2004 15314165 PMC506998

[B47] Vuoriluoto K Haugen H Kiviluoto S Mpindi JP Nevo J Gjerdrum C Tiron C Lorens JB Ivaska J Vimentin regulates EMT induction by Slug and oncogenic H-Ras and migration by governing Axl expression in breast cancer Oncogene 2011 30 12 1436 1448 10.1038/onc.2010.509 21057535

[B48] Hajra KM Chen DY Fearon ER The SLUG zinc-finger protein represses E-cadherin in breast cancer Cancer Res 2002 62 6 1613 1618 11912130

[B49] Sakai D Suzuki T Osumi N Wakamatsu Y Cooperative action of Sox9, Snail2 and PKA signaling in early neural crest development Development 2006 133 7 1323 1333 10.1242/dev.02297 16510505

[B50] Zhao JJ Lin J Yang H Kong W He L Ma X Coppola D Cheng JQ MicroRNA-221/222 negatively regulates estrogen receptor alpha and is associated with tamoxifen resistance in breast cancer J Biol Chem 2008 283 45 31079 31086 10.1074/jbc.M806041200 18790736 PMC2576549

